# Assessing Climate Change Impacts on Island Bees: The Aegean Archipelago

**DOI:** 10.3390/biology11040552

**Published:** 2022-04-02

**Authors:** Konstantinos Kougioumoutzis, Aggeliki Kaloveloni, Theodora Petanidou

**Affiliations:** 1 Laboratory of Botany, Department of Biology, University of Patras, 26504 Patras, Greece; 2 Laboratory of Biogeography and Ecology, Department of Geography, University of the Aegean, 81100 Mytilene, Greece; a.kaloveloni@aegean.gr (A.K.); tpet@aegean.gr (T.P.)

**Keywords:** biodiversity conservation, climate change, extinction risk, GIS analysis, Greece, Mediterranean, pollinator distribution, species distribution modelling

## Abstract

**Simple Summary:**

In this study, we conducted, for the first time, an extensive climate change impact assessment of bee pollinators in the Aegean Islands, Greece, a regional bee hotspot in the Mediterranean. We located the current biodiversity and future extinction hotspots in the region and identified areas in urgent need for conservation prioritization, by undertaking an overlap analysis with the established protected areas network in Greece. Most bee species occurring in the archipelago are expected to face severe range contractions and there is evidence of an underlying extinction debt in the study area. Our work could serve as the baseline for the integration of a rather neglected, yet extremely economically and ecologically important taxonomic group, the bees, in the systematic conservation planning in the archipelago.

**Abstract:**

Pollinators’ climate change impact assessments focus mainly on mainland regions. Thus, we are unaware how island species might fare in a rapidly changing world. This is even more pressing in the Mediterranean Basin, a global biodiversity hotspot. In Greece, a regional pollinator hotspot, climate change research is in its infancy and the insect Wallacean shortfall still remains unaddressed. In a species distribution modelling framework, we used the most comprehensive occurrence database for bees in Greece to locate the bee species richness hotspots in the Aegean, and investigated whether these might shift in the future due to climate change and assessed the Natura 2000 protected areas network effectiveness. Range contractions are anticipated for most taxa, becoming more prominent over time. Species richness hotspots are currently located in the NE Aegean and in highly disturbed sites. They will shift both altitudinally and latitudinally in the future. A small proportion of these hotspots are currently included in the Natura 2000 protected areas network and this proportion is projected to decrease in the coming decades. There is likely an extinction debt present in the Aegean bee communities that could result to pollination network collapse. There is a substantial conservation gap in Greece regarding bees and a critical re-assessment of the established Greek protected areas network is needed, focusing on areas identified as bee diversity hotspots over time.

## 1. Introduction

Hymenoptera, and more specifically, bees, are one of the largest and the most important insect groups, providing valuable ecosystem services, as they pollinate several economic crops and their wild relatives [1,2]. Thus, they have an important role in primary production and plant reproduction, in agribusiness and the global food-chain, and in nutrient-cycling and herbivory among others [3,4,5,6].

Nearly 20% of the IUCN assessed insects are currently threatened with extinction [7], including 9.2% of the European bees [8], mainly due to land-cover/land-use change, pesticide misuse, light pollution, pathogen spillover, and human-induced climate change [9,10]. These threats have been accelerating at an alarming degree over the past two centuries, leading to elevated extinctions, well beyond the background rate [11]. Insect declines, either in species richness or abundance, have already been recorded at all scales in various regions [12,13], irrespective of whether the latter are under any protection status or not [14,15,16]. This phenomenon has already altered biodiversity patterns and led to biotic homogenization, with land-cover/land-use change being the main stressor until now [17,18,19,20,21], forcing specialists and generalists to contract and expand their range, respectively [22]. Climate change, however, is expected to take the lead in the foreseeable future [21,23,24] and could cause both bottom-up and top-down cascading extinctions [25], due to phenological mismatches, metabolic meltdown, disruption of mutualistic relationships, and common insect traits, such as ectothermy and specialization ([21] and references therein). This would be more likely in islands, which usually are both biodiversity and extinction hotspots [26], and are more susceptible to the threats posed by invasive species, such as competitive exclusion and niche displacement [12,27,28,29]. Biotic homogenization in island insect communities might be further exacerbated by intensive honeybee keeping [30,31,32,33,34,35,36], which could trigger pollination network collapse if climate variables dysregulate the network’s structure [37].

Several studies have dealt with the effects of climate change on the distribution of pollinators, with the focus being on mainland regions [14,18,38,39,40]. None, however, have ever investigated how island pollinators might respond to projected elevated desiccation stress due to increased aridity and reduced precipitation patterns. Greece is one of the most species-rich Mediterranean countries both for plants [41] and insects [42,43,44,45], due to its rugged topography and its ~8000 islands and islets of varying size and isolation degree. The vast majority of these islands are located mainly in the Aegean archipelago, one of the largest archipelagos globally ([46] and references therein) and an ideal system for testing ecological hypotheses. The Aegean archipelago has been experiencing intense anthropogenic impacts since prehistoric times that have shaped its biotic communities ([47] and references therein). The delayed urbanization rates (compared to other south European countries, such as Italy and Spain) recorded in Greece [48,49] and the extreme climate anomalies that are anticipated in the region in the coming decades [50,51] might nevertheless challenge the resilience of insect populations [23]. Aegean biodiversity and biogeographical patterns are rather well-resolved [31,52,53,54,55], yet the insect Wallacean shortfall still remains partly unaddressed, rendering us blind regarding the Aegean insect biodiversity hotpots and how these might fare during the ongoing and projected thermophilization observed in Europe and the Mediterranean [56]. To date, climate change impact assessments are scarce in Greece, focusing mainly on plants and on mainland insect groups [57,58,59,60,61,62,63,64,65], even though we are aware that Aegean island insect communities and pollination networks are driven by contemporary climate [37,52]. Hence, understanding how island insects might respond to rapidly changing climatic regimes and locating current biodiversity and future extinction hotspots may contribute to a higher degree of integrated and intricate systematic conservation and management planning in Greece. By doing so, we could efficiently channel the conservation efforts and funds in areas that might constitute future ‘death-zones’, which are currently rich in species that provide important ecosystem services and fall outside the established protected areas network in Greece (Natura 2000) that covers ca. 28% of its terrestrial domain [66]. This is even more pressing, taking into account that of the relatively few studies that have assessed the effectiveness of the Natura 2000 network in Greece [46,67,68,69,70,71,72], only one has incorporated insects (and more specifically, threatened insects; [71]) and two have evaluated the network’s efficacy in protecting Greek biodiversity hotspots (in plants; [46,72]). Considering the drafting of species and habitats’ action plans that are underway and the country’s obligation regarding Aichi Biodiversity Targets 12 and 15 (prevent extinctions and enhance ecosystem resilience; [73]), the time seems fitting for providing a baseline regarding the current and future state of the Aegean island pollinator fauna and evaluating the intensity of human impacts on these taxa.

In this paper we focus on bees, the most important pollinator group in Greece and the Mediterranean [74,75], exploring, for the first time, their future distribution vis-à-vis their present status in the Aegean Islands. This is the first study attempting to investigate the impacts of climate change on bees in this part of the world. We use species distribution models and data collected on the Aegean Islands and we aim to:

(1) Assess how climate change might influence the bee diversity patterns in the Aegean Islands
(2) Locate the bee species richness hotspots in the Aegean Islands
(3) Investigate whether these hotspots might shift in the future and
(4) Assess their overlap with the Natura 2000 protected areas network.

## 2. Materials and Methods

### 2.1. Occurrence Data

We obtained species occurrences from the most detailed, in spatiotemporal terms, insect databases available for the Greek territory, namely the Melissotheque of the Aegean and ALARM databases, comprising data mostly systematically collected within the projects ALARM (2004–2008) and POL-AEGIS (2012–2015) [76,77]. Both databases together comprise 191,483 occurrences for 3061 insect species. We used a subset of this dataset, focusing on the 1153 bees occurring on 48 Aegean islands (142,438 total records). Duplicate data were removed and then we followed the protocols suggested by [78,79] regarding the spatial thinning of our data, so as to avoid pseudoreplication and associated spatial sampling biases. This procedure reduced our initial dataset to 6289 records for 358 bee species, since any taxa that had less than 3 occurrences were discarded from any further analyses, following [80].

### 2.2. Environmental Data

We estimated baseline monthly climate data from 1981–2009 (since all of our occurrence data were collected post-1990) for our study area using altitudinal data extracted from WorldClim [81] at 30-sec resolution (which is equivalent to ca. 1 km) via ClimateEU v4.63 as laid out in [82,83,84]. Afterwards, we generated the standard 19 WorldClim bioclimatic variables, as well as 16 additional environmental variables using functions from the “dismo” 1.1.4 [85] and the “envirem” 2.2 [86] R packages, respectively. Finally, we generated five additional abiotic (topographical: aspect, heat load index, slope, topographic position index and terrain ruggedness index) variables based on the altitudinal data derived from WorldClim and the functionality of the “raster” 2.6.7 [87] and the “spatialEco” 1.2-0 R packages [88].

We generated data for three time-slices [i.e., averaged for the 2020s, 2050s and 2080s; [83]] for three different (CCSM4, HadGEM2, and an ensemble of 15 global circulation models) Global Circulation Models (GCMs) and two different Intergovernmental Panel on Climate Change scenarios from the Representative Concentration Pathways family: RCP 4.5 (mild scenario) and RCP 8.5 (severe scenario), as described previously.

Nine uncorrelated predictors (Spearman rank correlation <0.7 and VIF < 10; [89]) were retained in our analyses after assessing multicollinearity via the “usdm” 1.1.18 R package [90] in order to minimize model overfitting.

### 2.3. Species Distribution Models

We followed the modelling procedure suggested by [91,92,93] to model the realized climatic niche of our taxa, using the Random Forest algorithm with the ‘ecospat’ 3.1 [94] R package. We created several sets of taxon-specific pseudo-absences according to the suggestions of [95,96] at a minimum distance of 5.5 km from presence locations, which equals the median autocorrelation distance among the non-collinear environmental variables, using functions from the ‘blockCV’ 1.0.0 R package [97]. We split ten times our data into training and testing sets (80:20 ratio) and then we evaluated our models’ performance using various metrics [98,99,100,101,102] using functions from the ‘CalibratR’ 0.1.2, ‘DescTools’ 0.99.40, ‘ecospat’ 3.2, ‘enmSdm’ 0.5.3.2, ‘Metrics’ 0.1.4, ‘MLmetrics’ 1.1.1 and ‘modEvA’ 2.0 R packages [103,104,105,106,107,108,109]. Finally, we compared our models against null models following [110].

We reconstructed the potential suitable area of each taxon for every time-slice using ensemble models [111], based on excellent-calibrated Ensemble of Small Models (TSS ≥ 0.8). The TSS score of each model was used as weight for each model’s contribution to the ensemble projection.

We used the metric that maximizes the sum of sensitivity and specificity [112,113,114] to generate binomial presence/absence maps for each Global Circulation Model, Representative Concentration Pathway and time-slice combination. We also nullified the suitability of any cells that had non-zero values in the clamping mask, to be more conservative in our predictions [115].

The ‘BIOMOD_RangeSize’ function from the “biomod2” 3.3.7 R package [116] helped us assess the direction (contraction or expansion) and magnitude of the range shift of all the taxa included in our analyses. All taxa were not assumed to have unlimited dispersal capacity, since this would be overoptimistic.

We assessed environmental extrapolation by estimating the ExDet metric and the proportion of data nearby in multivariate environmental space (%N) using functions from the ‘dsmextra’ 1.1.4 R package [117,118]. As some taxa are distributed beyond our study area, we also assessed if their niche was truncated using functions from the ‘humboldt’ 1.0.0.420121 R package [119].

All analyses were run in R 4.0.3 using base R functions and functions from “biomod2” 3.3.7 and “ecospat” 3.1 [94,116].

### 2.4. Biodiversity Hotspots Detection

We followed the rationale of [46] as laid out there, for all spatial analyses regarding species richness (SR) and corrected-weighted endemism (CWE; a geographically-weighted variant of SR; [120,121]) patterns. We defined L1 hotspots as the cells falling into the 1% quantile for both metrics following [122]. In other words, L1 hotspots are the 1% of the cells that have the highest score for each of the indices included in our analyses (i.e., SR and CWE). We used functions from [123] and the ‘phyloregion’ 1.0.4 R package [124,125,126] to estimate CWE and locate the L1 hotspots, respectively. L1 hotspots as herein outlined, come under the regional hotspots according to [127].

We assessed via Kruskal-Wallis and Watson tests, if the distribution centroids of the L1 hotspots might experience a spatiotemporal and altitudinal shift using base R functions. We used functions from the ‘sampbias’ R package [128] to assess the geographical accessibility bias in our dataset. Finally, we used the Global Human Modification Index [129], as a proxy of human impacts on current and future hotspots.

### 2.5. Protected Areas Overlap

To assess the effectiveness of the established protected areas network in Greece, we first retrieved data from the World Database on Protected Areas using functions from the “wdpar” 1.0.0 R package [130]. We then overlapped current and future L1 SR and CWE hotspots with the Greek protected areas network using functions from the “sf” 0.8.0 [131] R package. We restricted our overlap analysis to terrestrial Greece.

## 3. Results

### 3.1. Species Distribution Models Perfomance

**Figure 1:**
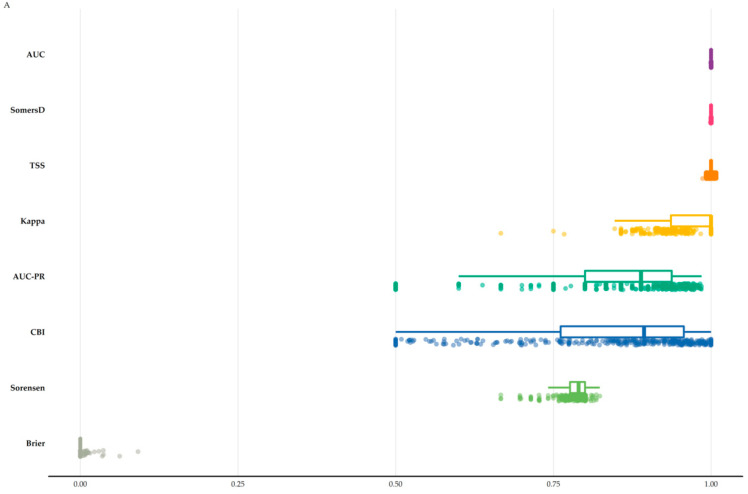

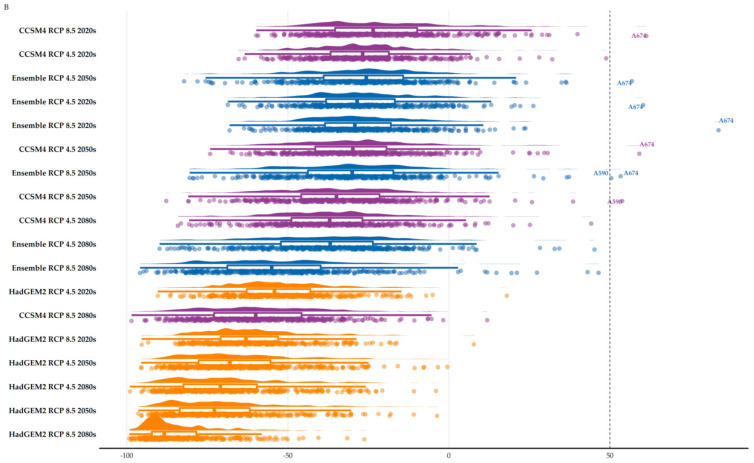
Raincloud plot of the (**A**) metrics to evaluate the models’ performance for all the taxa included in our analyses and (**B**) projected proportion of area range loss for all the taxa included in our analyses under any global circulation model (GCM) and representative pathway concentration (RCP) combination for every time-slice. AUC: Area under the curve. AUC-PR: Area under the precision-recall curve. CBI: Continuous Boyce Index. TSS: True Skill Statistic. A590 and A674 denote *Hoplitis fabrei* and *Melecta tuberculata*, respectively.

All models had high predictive power (Table S1; Figure 1A) and performed better than random at *p* < 0.01. Most of the studied taxa (90.22%) had low potential niche truncation index values (Table S1). Potential evapotranspiration of the coldest quarter (160 taxa) and precipitation of the coldest quarter (80 taxa) had the highest contribution among the environmental variables for most taxa, followed by aridity (44 taxa), while topographical variables emerged as the most significant predictors for 12.1% of the studied taxa (Table S2; Figure S1). Extrapolation novelty was negligible for all Global Circulation Models/Representative Concentration Pathways and time-slices, as the proportion of analogue climate ranged between 94.45–99.88% (Table S3; Figure S2). The proportion of data nearby in the multivariate space was generally high in most of the Aegean Islands under any uncertainty source (Figure S3). We found little variation among Global Circulation Models/Representative Concentration Pathways and the three different time-slices, and as such, we focus on the CCSM4 Representative Concentration Pathway 8.5 combination for the 2080s hereafter (the trends are similar among all uncertainty sources and they are steadily deteriorating across the three time-periods).

### 3.2. Area Range Change

There is some interspecific variation across all uncertainty sources regarding the direction and magnitude of the projected range shift, but most taxa are expected to experience range contractions, with these contractions gradually becoming more prominent in the long term (Table S4; Figure 1B). HadGEM2 had the highest mean range contractions under any Representative Concentration Pathway and time-slice (Table S4; Figure 1B), while the different bee families displayed the same temporal negative range change trend, which is expected to become more pronounced during the 2080s (Figure S4). Only two species, *Hoplitis fabrei* and *Melecta tuberculata*, are projected to experience substantial range expansions (>50%) during the 2020s and 2050s (Figure 1B).

### 3.3. Biodiversity Hotspots

**Figure 2:**
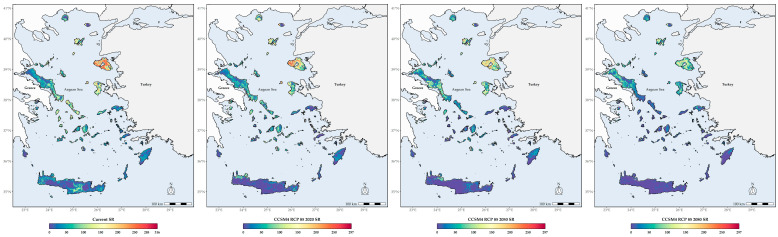
From left to right: Current bee species richness (SR) and future SR for the 2020s, 2050s and 2080s based on the CCSM4 8.5 Global Circulation Model/Representative Concentration Pathway combination.

**Figure 3:**
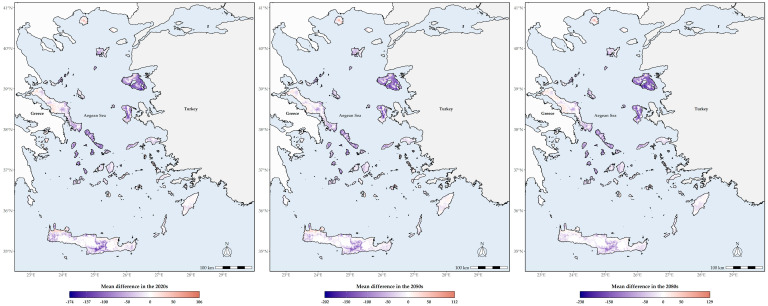
Mean difference between future species richness based on the CCSM4 Representative Concentration Pathway 8.5 for the 2020s, the 2050s and the 2080s, and current species richness.

**Figure 4:**
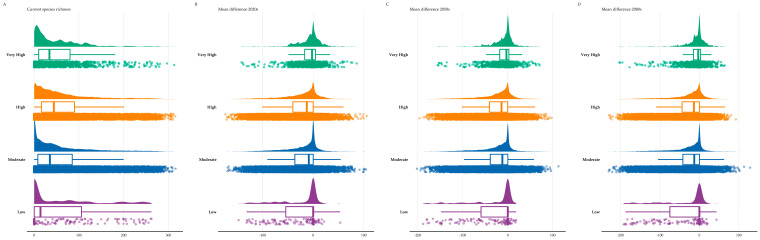
Raincloud plot of species richness and mean difference values observed in each of the four intensity classes of human impact in the Aegean Islands for the (**A**) current time period, (**B**) 2020s, (**C**) 2050s and (**D**) 2080s under the CCSM4 Representative Concentration Pathway 8.5 combination.

Lesvos and Chios currently display the highest SR, followed by Andros, Tinos and some thickets in northern Thasos and Samothraki, being, in general, moderately high in the central Aegean islands and lower in the southern Aegean islands (Figure 2). In the future, SR is projected to be gradually lower across all Aegean islands, as many taxa are expected to become locally extirpated in the next decades, with some very restricted areas in Lesvos, Kriti, Evvia, Samothraki, Thasos, Santorini, Astypalaea and Kalymnos displaying high SR values in the coming decades (Figure 2 and Figure 3). The same trends are also largely observed regarding CWE, as western Thasos and northern Evvia, along with Lesvos and Skiathos, display the highest CWE values until the 2050s, while in the 2080s, Astypalaea, together with some coastal areas in Skyros and in south-eastern Kriti and Rodos, will have the highest CWE values (Figure S5). In general, current L1 SR and CWE hotspots are located in Lesvos (Figures S6 and S7). In the future, these will be mainly located in the central and southern Aegean islands, with Lesvos having fewer such areas over time (Figures S6 and S7). Areas with moderate to very high Global Human Modification Index values currently exhibit statistically significantly higher SR values compared to areas with low-human impact values, with the latter showing net zero median species richness loss over time (Table S5; Figure 4).

### 3.4. Altitudinal and Latitudinal Shifts

**Figure 5:**
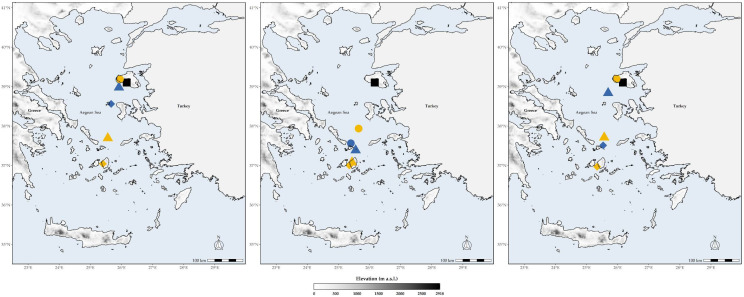
Distributional centroids for the L1 CWE hotspots. Black squares represent the distributional centroids of the current time-period. Circles, triangles and diamonds correspond to the distributional centroids of the 2020s, the 2050s and the 2080s time-period, respectively. The blue and yellow colours correspond to the Representative Concentration Pathways 4.5 and 8.5 across all panels, respectively. Left to right: CCSM4, Ensemble and HadGEM2 Global Circulation Model, respectively.

**Figure 6:**
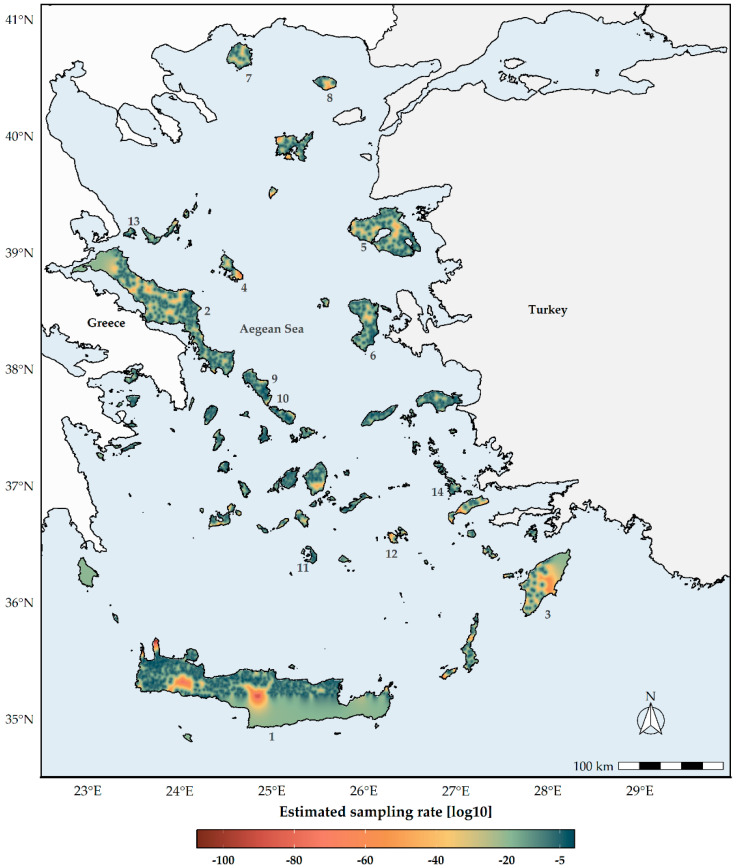
Log−10 transformed geographical projection of the sampling effort for the 358 bee species included in our analysis. Dark red cells denote underexplored areas. 1: Crete. 2: Evvia, 3: Rodos, 4: Skyros, 5: Lesvos, 6: Chios, 7: Thasos, 8: Samothraki, 9: Andros, 10: Tinos, 11: Santorini, 12: Astypalaea, 13: Skiathos, 14: Kalymnos.

The future L1 centroids for both SR and CWE are predicted to occur in lower latitudes and altitudes (Watson tests with *p*-values < 0.01 at α = 0.05; Kruskal–Wallis ANOVA: H = 1661.1, d.f. = 1026, *p* < 0.001; Figure 5 and Figure S8). Current L1 SR and CWE hotspots occur in higher altitudes, while future hotspots will be found at lower altitudes across all time-slices (Table S6). The sampling rate was mainly affected by the distance from cities/villages across the Aegean islands that we included in our analyses, while it was minimally affected by roads (Figure S9). The most underexplored and undersampled regions were the Cretan and Evvian highlands (Figure 6).

### 3.5. Protected Areas Network Overlap

Currently, 37–41% of the SR/CWE L1 hotspots are included in the Greek protected areas network and this proportion is expected to significantly decrease over time for both metrics, irrespective of the Global Circulation Model/Representative Concentration Pathway considered (Table S7).

## 4. Discussion

Greece and more specifically, the Aegean archipelago, is one of the most species-rich regions of the Mediterranean biodiversity hotspot regarding insects, including the main pollinator groups, viz. bees and hoverflies [42,43,44,132]. Despite the pollinators’ importance in crop quality and yield and the well-known negative impacts of anthropogenic disturbances on pollinator diversity and abundance, no study hitherto exists regarding the bee pollinators’ response to the ongoing climate change on island ecosystems. Here, we (a) assessed for the first time ever how climate change might affect island bee populations, (b) located the areas constituting current bee diversity hotspots in the Aegean archipelago, (c) checked whether these might experience spatiotemporal shifts in the future and (d) assessed the effectiveness of the Natura 2000 current network in protecting these areas over time. By doing so, we aim to lay out a baseline for the integration of a rather neglected, yet extremely important taxonomic group, namely bees, in the systematic conservation planning in Greece.

### 4.1. Climate Change Impacts

Insect populations, including pollinator groups, have been declining all over the globe, due to a multitude of stressors, the more pressing of which currently being land-use change [10,13,20]. Human-induced climate change on the other hand, is expected to become the major extinction driver in the coming decades [21]. These anthropogenic stressors have changed beta-diversity patterns [17], with many insect species facing dire future prospects [4,18,40,133]. Aegean bees do not seem to be an exception to this rule, as they are predicted to experience severe range reductions, with an elevated future extinction risk (Table S4; Figure 1 and Figure S4). We should note that there is however some interspecific variation in the species’ responses to changing climate regimes. This might be due to variation in physiological characteristics (e.g., thermo-and hydro-regulation ability; [133]), in species traits and adaptive plasticity [134] or in habitat specialisation. Regarding the latter, thermophilic species, nuisance pests and agricultural herbivores seem to fare better in a warmer world compared to cold-water, phryganic specialists or species with large home-ranges [12,135,136]. Only two species (Figure 1B), are projected to substantially increase their potential distribution and that not after the 2050s. Little is known regarding the ecology of these two species. *Hoplitis fabrei* is a range-restricted species occurring in Aegina and a few localities of the adjacent mainland (Petanidou, unpublished data; Devalez, personal communication). It is a rather thermophilic species specialising in plants belonging to the Boraginaceae family (Devalez, personal communication) encompassing mostly perennial plants covering a large part of the main flowering season. This kind of specificity may, or at least partly, explain (or sustain) the anticipated expansion of *Hoplitis fabrei* in the near future. *Melecta tuberculata* is a kleptoparasite of Anthophorini, which also visits a variety of plants belonging to very large families [137], such as Lamiaceae and Asteraceae, two of the most species-rich and nectar-rich families in the Aegean (Petanidou, unpublished data of the *Melissotheque of the Aegean* database). This implies that *Melecta tuberculata* participates in large pollination networks and can be considered as a generalist regarding its pollinating behaviour [137]. As most of the plants it can potentially feed upon will most likely be quite abundant in the near future (even if some of them disappear from its current range, others will still remain), this species could thrive in a warming world, as our results suggest. Be that as it may, further research regarding their ecology is definitely warranted.

The considerable range losses anticipated in the Aegean could be attributed to the species’ intolerance to warming and more arid climates, an evolutionary trait common to bees. This evidently impacts the cold-adapted bumblebees [138], but thermophilic wild bees are not destined to escape either. This is because in the Aegean archipelago, an area that is quite warm already, wild bee diversity depends positively on precipitation and negatively on temperature, as previous studies have shown [37,52]. This implies that increasing aridity vis-à-vis temperature are probably going to have detrimental effects on wild bees, albeit their thermophilic character. The individualistic response to climate change has also been observed in other insect groups [64,139], in high-altitude occurring species [63] and in other regions as well [39,138,140,141,142], pointing that even phylogenetically close and ecologically similar species might differ in their vulnerability against climate stressors [143]. It might as well be that abrupt future temperature rise will exceed the species’ thermal tolerance and/or the species’ ability to track their niche, especially in areas with low environmental heterogeneity, such as low altitude Aegean islands. However, this notion should be taken with a grain of salt, since the Aegean highlands are currently rather underexplored and there might be available niche space for at least some bee species occurring in the Aegean (i.e., those species with high potential niche truncation index values; Table S1).

As intense the direct abiotic effects of climate change might be, its indirect effects on biotic relationships may magnify its inimical impact on insects. These indirect effects may lead to spatiotemporal and phenological mismatches ([21] and references therein), decrease host plant quality [144] and increase interspecific competition due to the introduction of alien species [28]. The latter phenomenon is most likely already happening in the Aegean as well, where several alien species have been recorded [145]. Climatic factors, and more specifically, mean annual temperature and precipitation have shaped bee diversity patterns and plant-pollinator networks in the Aegean [37,52]. Precipitation and temperature have an inverse relationship regarding bee diversity in the Aegean [52]. This is in line with our results, since the potential distribution of most species is mainly driven by precipitation- and aridity-related variables (Figure 2). This implies that even thermophilic bees might not be affected by temperature rise, but rather by the projected increased aridity in the Aegean Islands, not to forget drought effects on the bees’ plant partners. Thus, it is not unreasonable to assume that the ongoing climate change will not only push several insect species to local extinction, but will also alter the dominance patterns of pollinator communities in the Aegean. This will probably lead to phenological shifts, and further disrupt pollination networks. In all likelihood, the latter will become less specialised. This will therefore further contribute to the projected biotic homogenization in the Aegean, a phenomenon anticipated for Aegean plants as well [59].

What is especially worrisome though, is that many of these species occur in highly disturbed areas (Figure 4A), which usually have a dramatic effect on insect communities [146]. The resilience of species occurring in such degraded habitats will be put to the test due to the synergistic effect of climate change and land-use change [23]. Even though man has shaped the Aegean landscape to a large extent [147], agricultural intensification has very recent roots in the region [48,49]. This shift in farming style has forced insect populations to decline or even to become extinct in other parts of the world [148,149]. Given that no bee species have been recorded as extinct in Greece or in the Aegean archipelago, this alludes that there may be an underlying extinction debt lagging in the Aegean waiting to be paid. After all, island communities are more extinction-prone [26], due to their lower functional redundancy [150], and species facing recent anthropogenic disturbance seem to be more vulnerable to extinction threats [17]. This phenomenon has been observed in Greek plants as well [72]. This means that even if the absolute number of insect species that might become locally extinct is at the lower end of our predictions at first, it is quite likely that a wave of secondary and cascading extinctions [25] will follow, due to the emergence of an extinction vortex in the Aegean plant-pollinator communities, as a result of resource-driven co-extinctions [151]. This could lead to pollination network collapse [37] and could be further exacerbated by several factors, such as the:

(1) intensifying land-use/land-cover change ([48,49]; https://land.copernicus.eu/pan-european/corine-land-cover (accessed on 17 February 2022)),
(2) increased aridity and subsequent desiccation stress,
(3) phenological mismatches between pollinator activity and pollinated plants,
(4) increased competition due to limited resources and the increased occurrence of invasive species, and finally,
(5) increased pesticide use [12,27,28,29].

The socioeconomic ramifications for the Greek agricultural production could be considerable, especially for those species that pollinate important crops [152], given that the ecosystems services stemming from pollinators are highly valuable (ca. US $ 577 bn/annum; [153]) and the agricultural sector comprises 4.1% of the Greek Gross Domestic Product [154].

### 4.2. Species Richness Hotspots

Several Aegean islands have emerged as biodiversity hotspots for different taxonomic groups [52,53,55,155,156,157,158,159,160,161]. Some southern Aegean islands, such as Kriti and Karpathos, are standing out for their endemic richness, while other Aegean islands, mainly from the archipelago’s eastern part, host a greater number of native species, possibly due to a rescue-area effect. The Aegean’s complex paleogeographical history, together with island area [162,163], have largely shaped these patterns, while precipitation and temperature seem to drive plant and bee diversity in the central Aegean islands [52,53]. Human disturbance has also forced several species to occupy nearly inaccessible cliffs and this will most likely intensify in the foreseeable future ([53] and references therein). Our results are in line with the aforementioned studies, since two eastern Aegean islands, namely Lesvos and Chios, emerged as L1 SR and CWE hotspots (Figure 2 and Figures S5–S7).

### 4.3. Altitudinal and Latitudinal Shifts of Species Richness Hotspots

We expect a latitudinal and altitudinal shift regarding the location of these hotspots over time. The central Aegean islands, which currently have lower than expected bee species [52], will serve as L1 hotspots for both metrics (Figure 2, Figure 3 and Figures S5–S7), but with substantially fewer species than currently observed (Figure 5). These will occur in lower altitudes (Table S6), due to the anticipated local extinctions (Table S4), as a result of increasing aridity and limited water availability (Table S1; Figure S1). However, we caution against these results, since the Aegean highlands are rather underexplored compared to the rest of the study area (Figure 6). At least the high-altitude areas in Thasos and Samothraki, the two northernmost Aegean islands, could buffer the effects of climate change, since topographically complex mountains might be environmentally heterogeneous enough for microclimatic refugia to occur, as is the case for Mt. Olympus [63]. On the other hand, the Cretan highlands most probably will not serve that purpose, as they are forecasted to become extinction hotspots in the future, at least for plants [59].

### 4.4. Conservation Implications-Assessment of the Effectiveness of the Greek Protected Areas Network

Bees constitute an integral component of natural and agricultural ecosystems [164], as they pollinate ca. 90% of the main global cash crops [5]. Thus, undertaking climate change forecasts for bees could be useful in mitigation action and conservation planning to avert any future food-chain imbalances [17] that might entail significant ecological and socioeconomic risks [3]. These imbalances could arise arising from the local, regional or even global extinction of important cash crop pollinators, such as bees.

Spatial conservation prioritization schemes often rely on the identification of regional biodiversity hotspots [121,165], thus allowing the design of cost-effective conservation management plans when limited-economic-and/or personnel-wise-funds are available [166]. Despite covering up to ca. 28% of the Greek territory [66], the Greek protected areas network includes no more than 41% of the Aegean L1 bee SR/CWE hotspots and this proportion is projected to decrease over time (Table S7). This overlap is lower than the one reported for the threatened insects occurring in Greece for any Aegean administrative region [71] and significantly lower than the one reported for Greek endemic plants [46]. This means that the conservation gap in Greece regarding bees is substantial and conservation actions are urgently needed if we are to halt the expected biodiversity declines in the Aegean. Our results lend further weight to recent studies suggesting a critical re-assessment of the established Greek protected areas network [46,71,72] and its potential expansion via the establishment of Key Biodiversity Areas or other effective area-based conservation measures [167,168], which are included in the post-2020 agenda [169]. The focus should thus be on the areas identified as bee diversity hotspots across time, which are not included in the Greek protected areas network since these areas might constitute climate refugia [39]—at least for the bees occurring in the Aegean archipelago. This line of thought results in the protection of as many species as possible, in an environmentally resilient, yet limited spatial extent that could be economically viable. In the meantime, less ambitious actions could be taken at the local scale, such as intercropping, use of selective pesticides, and attempts to actively involve stakeholders in landscape restoration and management [39,170], with land protection being however the most important conservation measure for insect conservation [20].

## Data Availability

Not applicable.

## Supplementary Materials

The following supporting information can be downloaded at: https://www.mdpi.com/article/10.3390/biology11040552/s1, Figure S1: Proportion barplot of the most important predictor variables. AIT: Thornthwaite’s Aridity Index. HLI: Heat Load Index. ISO: Isothermality. PCQ: Precipitation of the coldest quarter. PETCQ: Potential evapotranspiration of the coldest quarter. TPI: Topographical Position Index; Figure S2: Extrapolation novelty assessment based on the ExDet metric for the CCSM4 and RCP 8.5 combination in the (A) 2020s, (B) 2050s and (C) 2080s. All panels: Dark blue cells correspond to Type 1 novelty. Panels (B,C): green cells indicate areas with similar environmental conditions between the current and the future climate; yellow cells correspond to Type 2 novelty. Panel (A): yellow cells indicate areas with similar environmental conditions between the current and the future climate; Figure S3: Proportion of data nearby in multivariate environmental space for the CCSM4 and RCP 8.5 combination in the (A) 2020s, (B) 2050s and (C) 2080s; Figure S4: Raincloud plot of the projected proportion of area range loss for five of the bee families included in our analyses under CCSM4 RCP 8.5 for the (A) 2020s, (B) 2050s and (C) 2080s. Mellitidae are not presented as they comprise only one taxon; Figure S5: From left to right: Current bee corrected weighted endemism (CWE) and future CWE for the 2020s, 2050s and 2080s based on the CCSM4 8.5 GCM/RCP combination; Figure S6: From left to right: L1 (top 1%) species richness hotspots (red cells) for the current and future species richness for the 2020s, 2050s and 2080s based on the CCSM4 8.5 GCM/RCP combination, respectively; Figure S7: From left to right: L1 (top 1%) CWE hotspots (red cells) for the current and future CWE for the 2020s, 2050s and 2080s based on the CCSM4 8.5 GCM/RCP combination, respectively; Figure S8: Distributional centroids for the L1 species richness hotspots. Colour circles represent the distributional centroids of the current (purple), the 2020s RCP 4.5 (violet), the 2050s RCP 4.5 (dark green), the 2080s RCP 4.5 (dark blue), the 2020s RCP 8.5 (yellow), the 2050s RCP 8.5 (light red) and the 2080s RCP 8.5 (olive green) time-period. Left to right: CCSM4, Ensemble and HadGEM2 GCM, respectively; Figure S9: Posterior weights for each of the bias factors. (B): Sampling rate change based on distance in km for each of the bias factors; Table S1: Evaluation of models’ predictive performance via several discrimination (AUC, AUC-PR, TSS) and calibration [Brier score, Cohen’s kappa, Continuous Boyce Index (CBI), Somer’s D] metrics based on a repeated (10 times) split-sampling (calibration data: 80%; evaluation data: 20%) approach. PNTI: Potential Niche Truncation Index; Table S2: Variable importance for each of the bee species occurring in the Aegean Islands and included in our analyses, along with the abbreviations of the predictor variables; Table S3: Extrapolation in environmental space estimation for each Global Circulation Model (GCM)/Representative Concentration Pathway (RCP) and time-slice combination. AIT: Thornthwaite’s aridity index. PCQ: Precipitation of the coldest quarter. PETCQ: Potential evapotranspiration of the coldest quarter; Table S4: Proportion of potential area loss for each of the bee species occurring in the Aegean Islands and included in our analyses for every time-period and climate change model/scenario. GCM: Global Circulation Model. RCP: Representative Concentration Pathway; Table S5: Median, mean, minimal and maximal species richness (SR) and mean difference values observed in each of the four intensity classes of human impact in the Aegean Islands for each GCM/RCP and time-period combination. GCM: Global Circulation Model. IQR: Interquartile range. RCP: Representative Concentration Pathway. SD: Standard deviation; Table S6: Median, mean, minimal and maximal altitude (in m) for species richness (SR) and the corrected weighted endemism (CWE) hotspots. L1 species richness (SR) and corrected-weighted endemism (CWE) hotspots are delineated as the cells belonging to the 1% quantile for each metric for each GCM/RCP and time-period combination. GCM: Global Circulation Model. IQR: Interquartile range. RCP: Representative Concentration Pathway. SD: Standard deviation; Table S7: Percent overlap (%) between the Protected Areas network (PA) in the Aegean Islands and the species richness (SR)/corrected weighted endemism (CWE) L1 hotspots identified in the present study. L1 hotspots refer to the 99% quantile. GCM: Global Circulation Model. RCP: Representative Concentration Pathway.
