# Supplementary material for: Assessing Climate Change Impacts on Island Bees: The Aegean Archipelago
Source: Biology (Basel). 2022 Apr 2;11(4):552. doi: 10.3390/biology11040552 (PMC9030098; doi:10.3390/biology11040552)
Supplement: Supplementary file 1 [file biology-11-00552-s001.zip › Supplementary Figures.pdf]

Climate change impacts on island bees: the Aegean archipelago

Biology

Supplementary figures

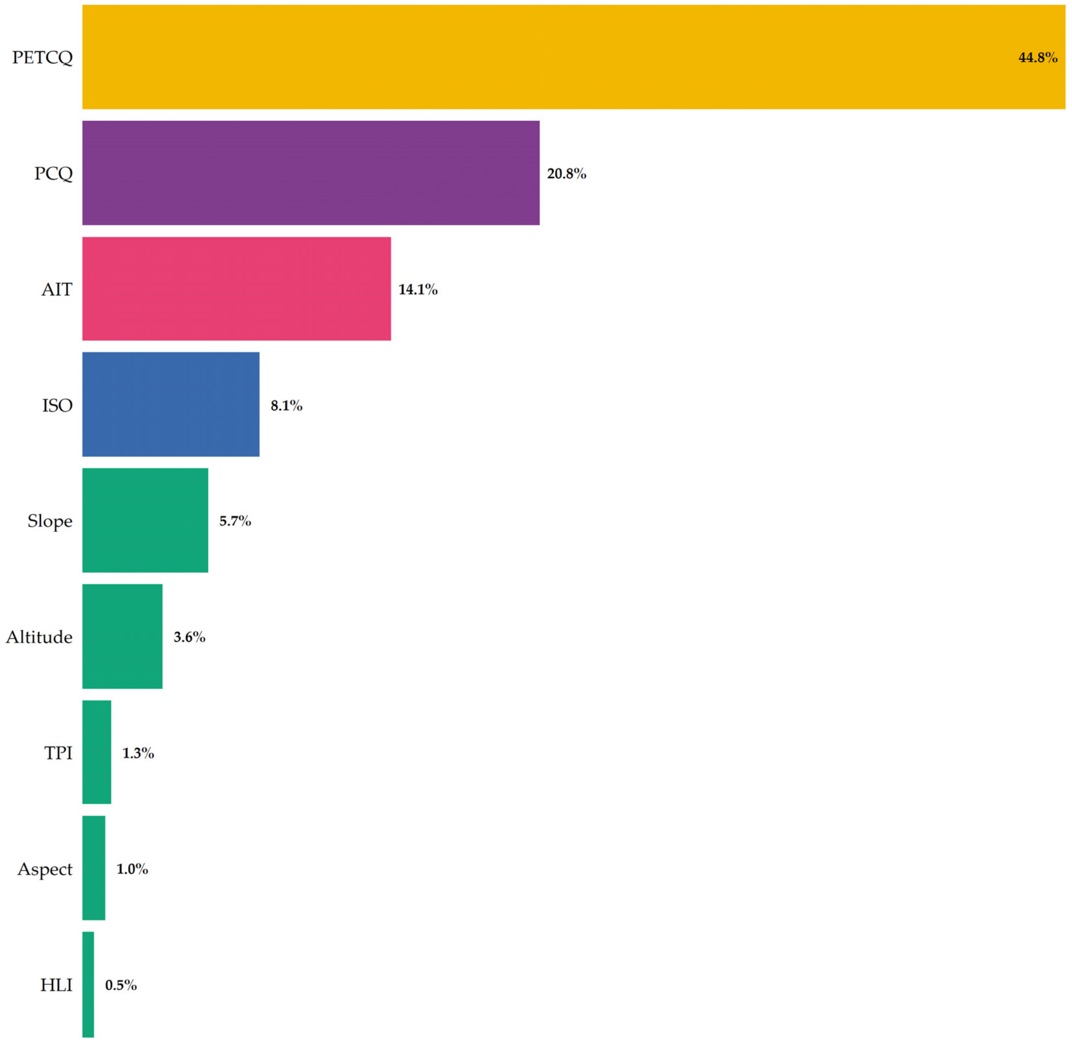

Figure S1. Proportion barplot of the most important predictor variables. AIT: Thornthwaite's Aridity Index. HLI: Heat Load Index. ISO: Isothermality. PCQ: Precipitation of the coldest quarter. PETCQ: Potential evapotranspiration of the coldest quarter. TPI: Topographical Position Index.

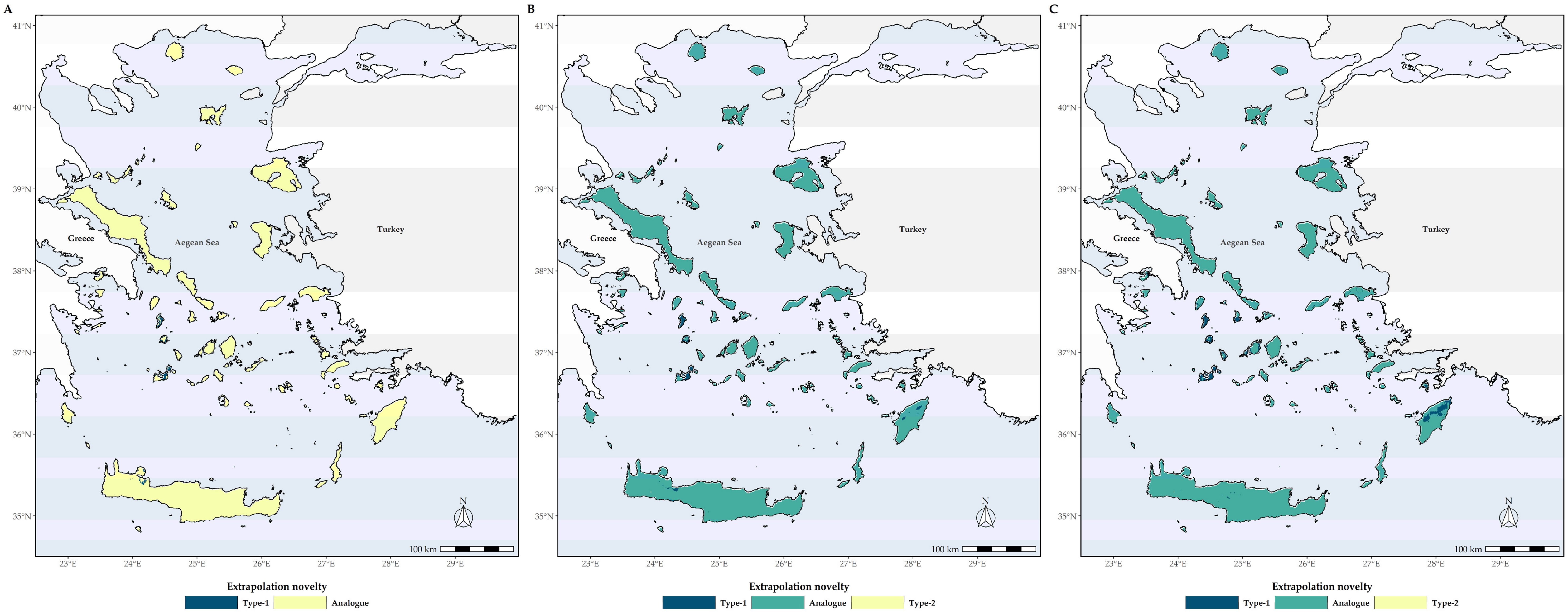

Figure S2. Extrapolation novelty assessment based on the ExDet metric for the CCSM4 and RCP 8.5 combination in the (A) 2020s, (B) 2050s and (C) 2080s. All panels: Dark blue cells correspond to Type 1 novelty. Panels (B) and (C): green cells indicate areas with similar environmental conditions between the current and the future climate; yellow cells correspond to Type 2 novelty. Panel (A): yellow cells indicate areas with similar environmental conditions between the current and the future climate.

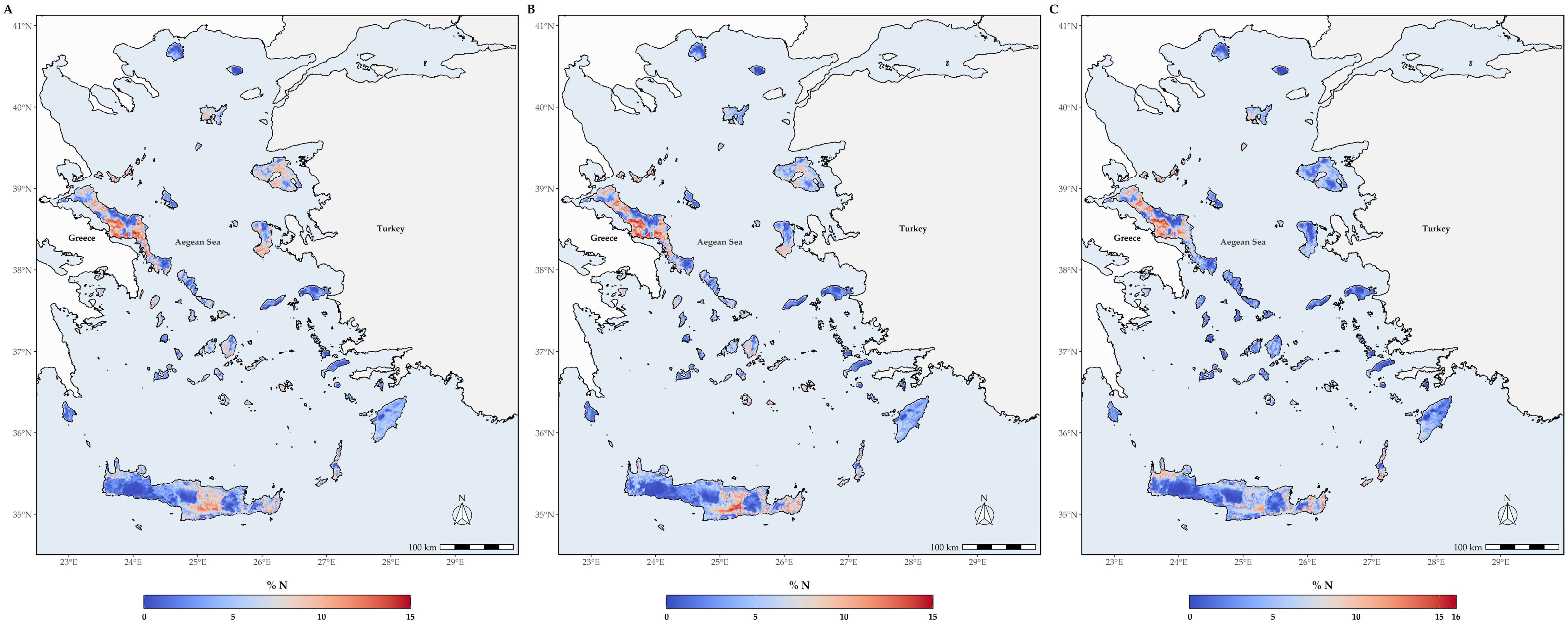

Figure S3. Proportion of data nearby in multivariate environmental space for the CCSM4 and RCP 8.5 combination in the (A) 2020s, (B) 2050s and (C) 2080s.

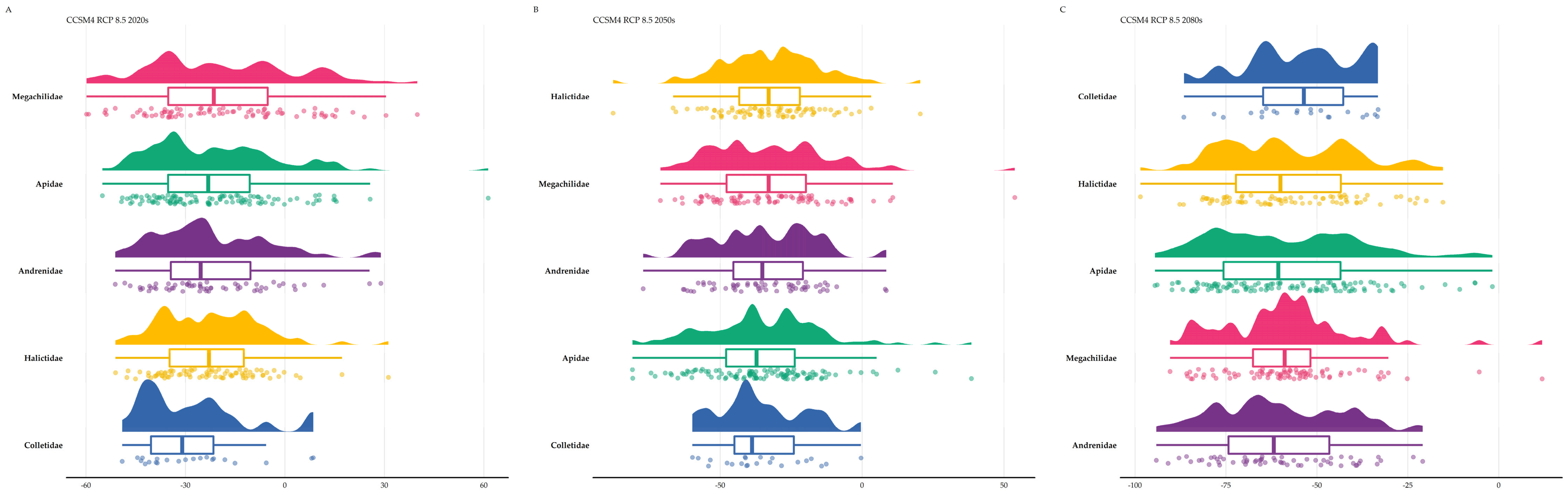

Figure S4. Raincloud plot of the projected proportion of area range loss for five of the bee families included in our analyses under any CCSM4 RCP 8.5 for the **(A)** 2020s, **(B)** 2050s and **(C)** 2080s. Mellitidae are not presented as they comprise only one taxon.

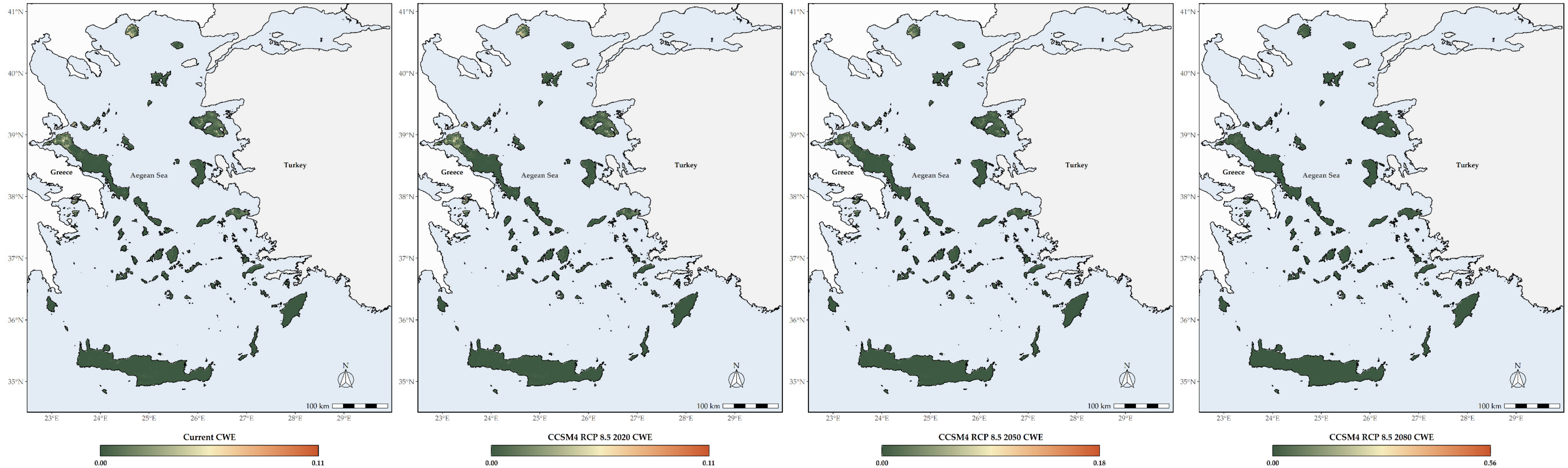

Figure S5. From left to right: Current bee corrected weighted endemism (CWE) and future CWE for the 2020s, 2050s and 2080s based on the CCSM4 8.5 GCM/RCP combination occurring in the Aegean islands.

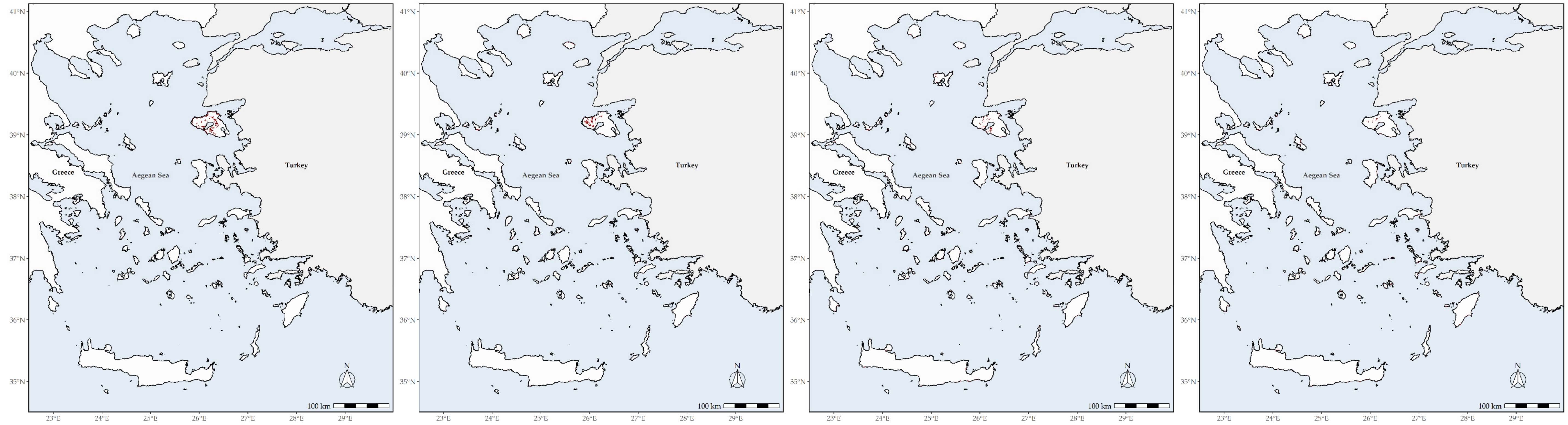

Figure S6. From left to right: L1 (top 1%) species richness hotspots (red cells) for the current and future species richness for the 2020s, 2050s and 2080s based on the CCSM4 8.5 GCM/RCP combination occurring in the Aegean islands, respectively.

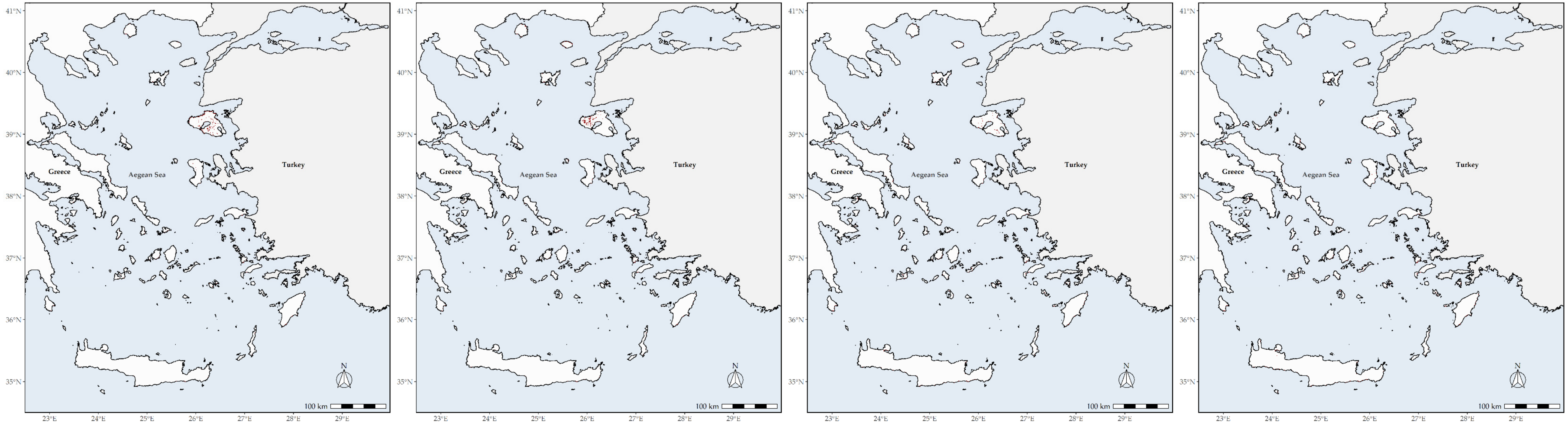

Figure S7. From left to right: L1 (top 1%) CWE hotspots (red cells) for the current and future CWE for the 2020s, 2050s and 2080s based on the CCSM4 8.5 GCM/RCP combination occurring in the Aegean islands, respectively.

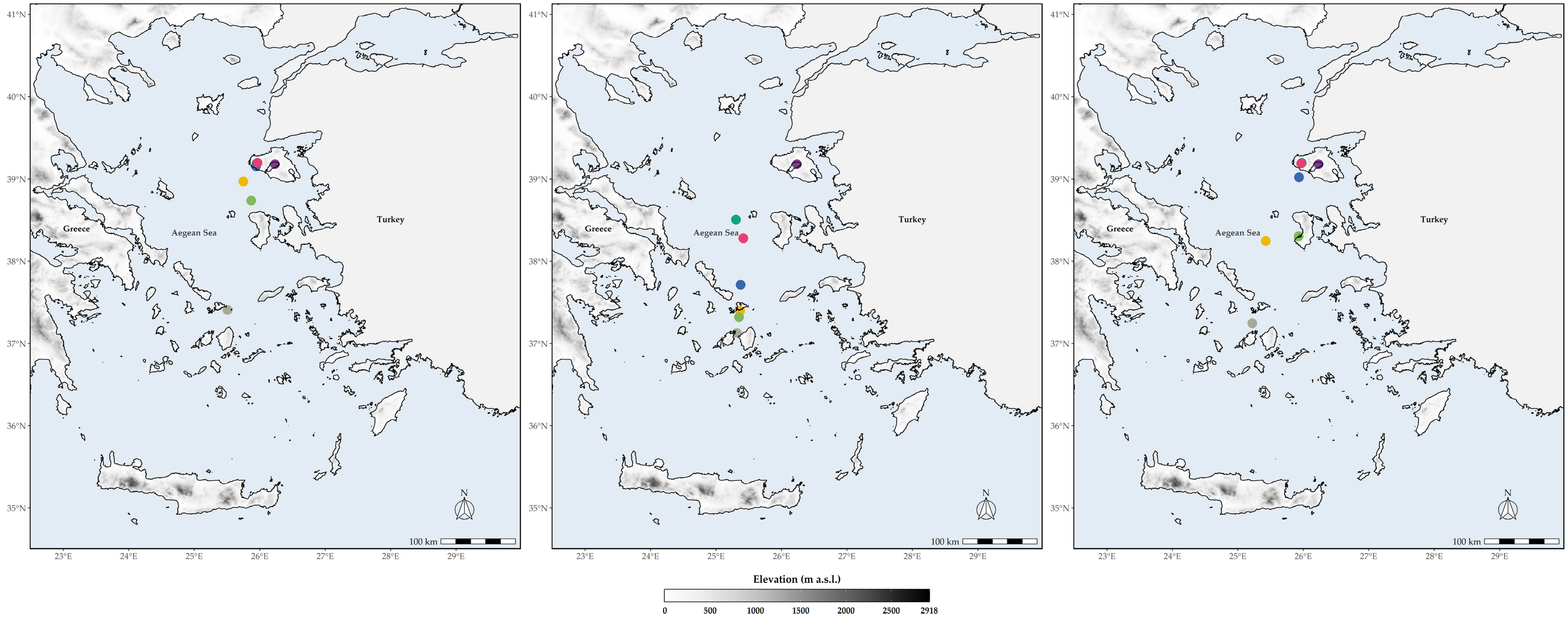

Figure S8. Distributional centroids for the L1 species richness hotspots. Colour circles represent the distributional centroids of the current (purple), the 2020s RCP 4.5 (violet), the 2050s RCP 4.5 (dark green), the 2080s RCP 4.5 (dark blue), the 2020s RCP 8.5 (yellow), the 2050s RCP 8.5 (light red) and the 2080s RCP 8.5 (olive green) time-period. Left to right: CCSM4, Ensemble and HadGEM2 GCM, respectively.

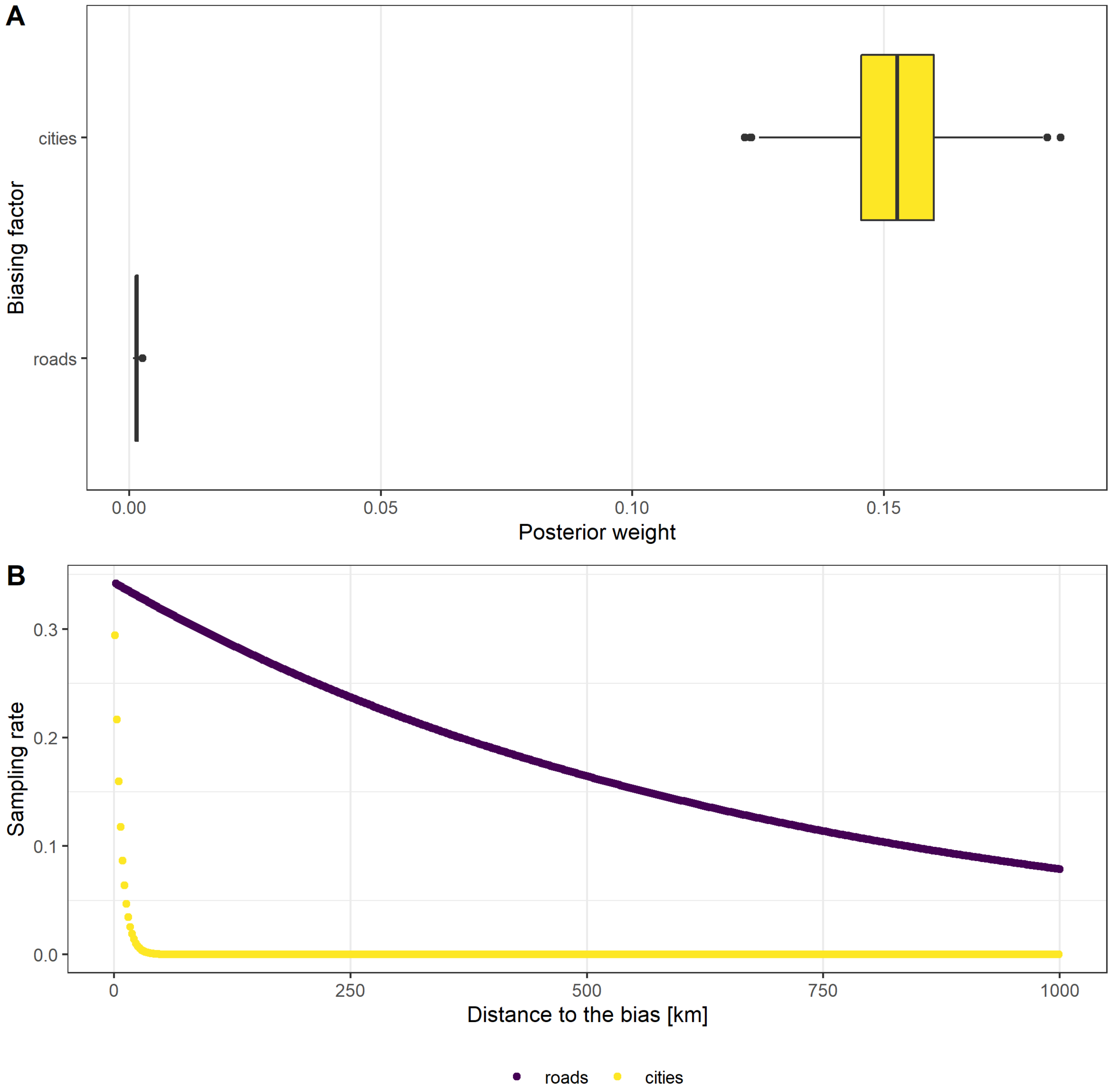

Figure S9. (A): Posterior weights for each of the bias factors. (B): Sampling rate change based on distance in km for each of the bias factors.
